# Business-life balance and wellbeing: Exploring the lived experiences of women in a low-to-middle income country

**DOI:** 10.3402/qhw.v11.30492

**Published:** 2016-04-12

**Authors:** Dorothy I. Ugwu, Charles T. Orjiakor, Ibeawuchi K. Enwereuzor, Christiana C. Onyedibe, Leonard I. Ugwu

**Affiliations:** 1 Department of Health and Physical Education, University of Nigeria, Nsukka, Nigeria; 2 Department of Psychology, University of Nigeria, Nsukka, Nigeria

**Keywords:** Work-life, business-life, balance, well-being, low-to-middle income

## Abstract

**Aim:**

With most studies on work-life balance focused on employees, this study sets out to explore the everyday living of business women who trade on petty goods and earn very little in a low-to-middle income country (LMIC). We explore their conceptions of balance, how they manage intersecting roles, and how they cope with daily hassles and stress to maintain wellbeing.

**Background:**

With the proportion of self-employed to employed people in Sub-Saharan LMICs being an inverse of the situation in Euro-American countries, there is a need to explore what balance could mean for the people in LMICs. Most studies in the work-life literature have explored how employees pursue balance and the various strategies that work for a specific group of people. Perhaps because work-life balance literature has largely sprung from advanced economies, little focus has been placed on how other societies, especially people in LMICs, navigate balance, given their unique milieu.

**Design:**

Adopting the reflective life-world approach, we inquire into the daily lives of women in very small businesses.

**Method:**

Twenty women who trade on a range of items and earn very little (gross daily sales of $0.41 to $62.98) were interviewed using a semi-structured guideline. Analysis was conducted using interpretative phenomenology.

**Result:**

Conceptions of balance for the women incorporated the notions of satisfactory progress across roles, proper time apportionment to roles, conditional balance as well as harmony and/or synchrony across roles—a slight difference from the popular understandings. Their conception of business life roles was deemed much more integral. Negative physical and psychological experiences impacting health and wellbeing, identified as culminating as a result of both roles, were commonplace but were typically considered a normal part of living. Engagements in extra-social roles appeared to have a double-edged effect. Placing the family first, time management, and prioritizing were some of the important measures of ensuring balance and wellbeing. Financial gains and personal satisfaction were top motivational reasons that kept the women committed to pursuing simultaneous roles.

**Conclusion:**

There is a strong overlap between what balance means for petty trading women and employees. However, the unique social platform offers a more communal perspective of issues in pursuing balance.

Concern for work-life balance and the impacts of imbalance continues to gather momentum in research. There is ample evidence that, on one hand, work-life imbalance impacts physical, mental and general health, and wellbeing (Carlson et al., 2011; Frone, 2000; Rantanen, Kinunnen, Feldt, & Pulkkinen, 2008; Shockley & Singla, 2011) and, on the other hand, work-life conflict undermines productivity and performance in the workplace (Zheng, Molineux, Mirshekary, & Scarparo, 2015). Consequently, organizational initiatives and government policies, at least in advanced Western economies, deploy means to foster work-life balance in their employees. Organizational initiatives take the shape of education programmes, leave policies, child care, telework, and other practices contributing to employees’ work-life balance. Government policies, which sometimes come in the form of employment guidelines and regulations, pressure organizations to conform to effecting direct and indirect betterment of work-life balance. Some evaluative studies have reported policy and initiative efforts to be helpful in promoting wellbeing in workers (Pitt-Catsouphes, Matz-Costa, & MacDermid, 2007; Zheng et al., 2015).

However, a disturbing bias can be observed in the trend of work-life research. The concept of “work” in work-life literature is largely tilted to understanding employees who work for private organizations or in the public sector. Yet employees are not the only genre of people who experience the need to balance their involvements in earning revenue and their not-in-work time. Individuals who run their own businesses to earn a living, commonly known as entrepreneurs, are not immune to difficulties in achieving wellbeing in their business engagements and fulfilling other-life commitments. A scan of the literature reveals a handful of studies exploring issues of balance in people running their businesses compared to a mammoth number of studies focusing on employee work-life wellbeing. Apparently, policies and work-place initiatives designed to enhance work-life balance largely reflect the needs of employees. Perhaps because entrepreneurship and self-employment offer autonomy and flexibility, it is assumed that entrepreneurs are at liberty to take leaves as desired or design their activities to optimally attend to work and other-life roles. Nonetheless, business people have been reported to experience more stress compared to organizational employees as they spend longer hours attending to increased responsibilities (DeMartino, Barbato, & Jaques, 2006). Prottas and Thompson (2006) equally found that the favourable outcomes associated with autonomy among the self-employed have more to do with the specific nature of the work and demographic characteristics.

As the word “work” in the phrase “work-life balance” has been largely used to connote employees, we decide to use *“*business-life balance” in discussing the experiences of individuals who are self-employed. By using the term business-life, we hope to focus on a specific category of people, not just people in work in general. It may, however, be argued that whether self-employed or employed by another party, activities relating to income generation for the individual may be referred to, in a general sense, as work. We hence will use “work-life” in discussing general issues in literature and “business-life” in addressing specific issues concerning self-employed people or entrepreneurs.

## The concept of work-life balance; business-life balance

Taking a closer look at the concept of balance in the four decades of work-life research, not neglecting the considerable influence in policy making in the work-life sphere, the scope, breadth, and depth of the work-life system is still inconclusive (Greenhaus, Collins, & Shaw, 2003; Munn, 2013). Clark (2000), for instance, highlights “satisfaction and effectiveness” in roles with minimal conflict to underscore balance. Marks and MacDermid (1996), on the other hand, view balance in the light of “equal attention” to both roles. Greenhaus et al. (2003) unified the two views, conceiving balance to run on a continuum including the extent of equal engagement as well as equal satisfaction in both roles; incorporating the time, involvement, and satisfaction dynamics of balance. Recent literature tends to consider work-life roles to be integrated, each role complementing the other (Greenhaus & Powell, 2006). More recently, Greenhaus and Allen (2011) have broadened the scope of the work-family concept to mean an assessment of the extent to which peoples’ effectiveness in work and family roles align with their life values in a given time. Terms such as rhythm (Hughes & Bozionelos, 2007), harmony (McMillan, Morris, & Atchley, 2011), and work-life system (Munn, 2013) are now used in the literature.

A holistic conception of work-life balance would consider the nature and dynamics of work/business, family systems, government and work place policy, economics, the business environment, individual personality configurations, values, geography, and possibly socio-cultural patterns (Crooker, Smith, & Tabak, 2002; Khallash & Kruise, 2012; Munn, 2013; Zuba & Schneider, 2013). Munn (2009) points out that a “one-size-fits-all” approach does little to foster the understanding and advancement of work-life balance for the wider society. Some studies reviewing programmes designed to tackle work-life conflicts reveal that some groups (e.g. high-mid placed salaried workers and traditional families, see Galinsky, 2009; Lambert, 2009; Munn, 2013; Williams, 2010) are favoured. Others (e.g. low -income and hourly paid workers and gay/lesbian employees, see Hornsby & Munn, 2009) come out with the shorter end of the stick, especially if other factors such as earning power, and child and elderly care are considered. Many researchers are of the view that solving the work-life conundrum is still far off (Munn, 2013).

Again, there is little in literature regarding how different categories of self-employed people fare regarding the business-life balance. One then wonders how government policies and corporate initiatives towards balance accommodate balance for the self-employed. Yet, entrepreneurs are a critical part of society and a nursery for the future corporations that employ others. It is therefore important that better and deeper attention be given to understanding how individuals who are self-employed accomplish business-life balance. Understanding business-life balance will extend and broaden the frontiers of exploration, adding more evidence-based findings into better ways to ensure and sustain balance for a healthy and happy working population. Also, what works for a specific group of people (e.g. entrepreneurs and employees) would be better understood.

## Theoretical framework

Contending contemporary theories of work-life balance and conflict involve two apparently opposing views. Marks's (1977) scarcity theory suggests that work and family roles compete for attention and resources, with one role intruding into the other, resulting in people not completely attending to both roles. A structured narrative review of the literature by Beauregard and Henry (2009) found that work-life conflict, indeed, had a negative impact on individuals. Alternatively, Greenhaus and Powell (2006) suggest that double (and maybe multiple roles) enrich and enhance individual functioning with the benefits of either role spilling over to compensate for the shortfalls of the other. Thus, proceeds from the business place help in providing for family needs and, conversely, success in catering for education needs, resulting in excellent academic success, boosts self-esteem and feelings of personal achievement.

One way to reconcile these opposing perspectives is to recognize that what constitutes stress differs among individuals, and people vary in the amount of stress they can tolerate. For example, Reid, Stajduhar, and Chappell (2010) concluded that work may provide relief for some people engaged in caregiving, while some may find employment adding to their stress. Also, some persons remain productive under stress. The assessment of situations is highly subjective. Yet another important theoretical underpinning is the social role theory that identifies the normative gender roles that individuals play.

## Women in work and business-life balance

About 70 and 60% of women in the developed and developing worlds, respectively, are reported to be actively engaged in work (International Labour Organisation, 2010). Their contributions to the economy globally are well acknowledged (Nel, Maritz, & Thongprovati, 2010). Women, specifically mothers, perhaps deserve better attention, and indeed have received considerable devotion in the work-life literature as they combine the arduous roles of work, and child and home care (Bird, 2006; Hochschild, 1989; Lewis, 2001; Munn, 2013). Indeed work-life balance literature is understood to be focused on reflecting and resolving the struggles of working mothers (Lewis, Gambles, & Rapoport, 2007). Studies have explored issues of work-life balance in Scandinavian single mothers (Bull & Mittelmark, 2009), Filipino and Pakistani women entrepreneurs (Edralin, 2012; Rehman & Roomi, 2012), and women software professionals in India (Valk & Srinivasan, 2011). The career trajectory of women and how it affects general wellbeing and satisfaction has equally been examined in the United Kingdom (Tomlinson, 2006).

From another perspective, issues in work-life balance may be understood to reflect cultural variations such that people's experience of the balance phenomenon can be understood to reflect the wider societal and cultural patterns in which they live. Rehman and Roomi (2012), for instance, identified that implicit socio-cultural norms significantly affect the dynamics of pursuing work-life balance in Pakistani women entrepreneurs. They repeatedly identified that the dynamics of balance may be different for Islamic women in Pakistan as a result of social expectations, specifically issues regarding family honour and *purdah*, pointing to the veiling or seclusion of women in active public life. Conversely, Tomlinson (2006) found that work and employment trajectories, as well as work-life choices for women in the United Kingdom, were influenced by care networks, work status, welfare policies, and also individual preferences. This points to the fact that the concept of balance and its pursuit, indeed, can mean different things for different persons in different places. Lewis et al. (2007) identified that concern for balance in the work-life literature is higher for mothers in societies with traditional gender value roles. Also, research has identified women in low-skilled, low-status jobs as well as low-income earners and ethnic minorities to be heavily impacted by the pressures of the work/business, and family care (Austen & Ong, 2014; Feinberg & Choula, 2012). Here, we focus on the issue of balance among women who earn low incomes in the African continent, a group poorly represented in the work-life literature, exploring how their experiences fit into the work-life literature. We pay attention to how much, and in what manner their local environments have shaped their experiences.

## Work/business-life balance in Africa

Using the World Bank classification of countries on the economics and wealth, most African and Sub-Saharan countries are in the lower- to middle-income categories, with GDPs within the range of USD1006 to USD12,275 on the low-income to the upper-middle-income band (Gindling & Newhouse, 2013). According to a World Bank report, in low-and middle-income countries (LMIC) of the globe, less than half of the population (49.3%) are on paid wages or salary, and most are self-employed (Gindling & Newhouse, 2013). The percentage of people in paid employment drops to only 17% for people in Sub-Saharan Africa. The difference is large when compared to the over 85% of people in paid employment in European countries (Hatfield, 2014). From the indices above, about 80% of the people in Sub-Saharan Africa engage in some kind of self-employment to sustain their livelihoods. Clearly, this is the direct inverse of the Euro-American situation, in which over 80% of the population are employees. This gives a hint as to why the work-life literature has largely focused on the employee population, as work-life study has been largely pursued by people from the neoliberal economies (Lewis et al., 2007). It is hence possible that the dynamics of the work/business-life for people in LMICs may indeed be different, and even more different for people in Sub-Saharan Africa.

With little to no welfare state in the region, the difficulty is compounded by the absence of measures to cushion the stress of work/business-life roles. People in this category are very unlikely to have business-place benefits, including maternity/paternity leave and child care provisions. The majority are low-wage earners and little is understood about the work-life balance among low-wage earners who do not receive welfare (Munn, 2013; Seefeldt, 2008). Many carry their sale wares and produce to the home and back to the business place; their businesses operate at least 6 days of the week, including on national holidays; and there is little to no government support towards their wellbeing.

Despite these difficult conditions identified above, much less attention is given to understanding what balance fundamentally means for some others in the developing world (Aryee, 1992). Research from the African continent is sparse in the work-life literature, meaning that the peculiarities of the continent have had little influence in the work-life literature. The handful of studies exploring work-life balance in Africa have mostly conformed to American and European studies by exploring the concept among employees, most of whom work in public offices or for private firms. There is need, therefore, to explore more aggressively the issue of balance for self-employed individuals in the Sub-Saharan region, given the demography of the region.

The importance of intensifying work/business-life research in Africa is even more important considering a unique social system, culture, and lifestyle distinct from the West from which the work-life literature sprang. Eby, Casper, Lockwood, Bordeaux, and Brinley (2005) identified that it is insufficient to focus solely on the work and family domains if we aim to understand how individuals balance different roles. Also, Kreiner, Hollensbe, and Sheep (2006) opined that individual identities, defined as the aspects of self that arise from personal characteristics and the social categories in which the individual claims membership, should be incorporated in the interface across life domains. Social engagements have a special place in the milieu of most African cultures. This comes from commitments in the traditional, religious, and closely knitted family system practices commonplace in the region. It has been reported that these practices offer unique support structures that cushion the effects of stress (Ugwu, 2010; 2014). Nevertheless, attending to these social engagements, especially when considered in the mix of family and business needs, can add to the negative stressful experiences of individuals. Hence, social engagements may influence the behaviour of people in both family and work domains. To this effect, the unique contribution of this study is the focus on the type and nature of business-life conflicts experienced, and how people balance the conflicting role pressures from different domains, including the demands emanating from social engagements.

Our aim in this paper is to qualitatively explore the everyday lives of self-employed women living in a Sub-Saharan LMIC; focusing on how they conceive balance, navigate overlapping business and life roles, and cope with the daily challenges of combining business with their other roles in order to maintain wellbeing. Nigeria, the setting of the study, is the most populous country in Africa, the sixth most populous country in the world, and is considered hugely important to the advancement of the continent.

## Method

### Phenomenological design

Most researches in work-life balance are largely quantitatively styled, adopting a nomothetic style in investigating issues of conflict and balance among working people. Quantitative methodology is less fitting for an exploration of the topic in a region with little media emphasis and publicity on the issue, and relatively low literacy levels. Therefore, to explore people's experiences regarding business-life balance in everyday life, a phenomenological design was adopted for this study. Existential phenomenology offers a unique approach that gives human experiences and their meaning a centre place (Heidegger, 1962). The phenomenological approach strives at rendering a true-to-life description of participants’ experiences, highlighting predominant themes embedded on the four existential grounds of the body, time, others, and the world (Thomas, 2003). In this study, we adopt Merleu-Ponty's philosophy of phenomenology, but specifically, Smith and Osborn's (2008) interpretative phenomenology analysis (IPA) in looking into the experiences of women petty traders. IPA commits to a person's cognitive, linguistic, affective, and physical being, assuming a link between cognition and emotion in individuals (Smith, 2004; Smith & Osborn, 2008). IPA gives flexibility by staying open to theory and literature, but also to new ideas and clusters of meaning (Maxwell, 2005; Miner-Romanoff, 2012). Furthermore, IPA adopts the double hermeneutics approach in which the participants try to make meaning of their world, and researchers try to understand this meaning as well as their place in theory.

### Participants

Twenty women who trade on a range of items participated in the study. They were purposively selected from a local market in a city in South-Eastern Nigeria. Their age ranged between 27 and 64 with a mean age of 34.7 years. Respondents were typically married (one was a widow), reported having a range of none to seven children, lived with at least one elderly person for whom they provided care, and had a family size of two to nine people. Participants, by and large, sell items such as fruits and vegetables, spices, nuts, fish, and general farm produce. Others were a food vendor, hair stylists, and others who traded on confectionery and clothings/beddings on a small scale (see Table I). Most had their wares displayed on a table with an umbrella for shade, others displayed their goods on wheel barrows. Two participants (with long-standing businesses in the area) were operating from small stalls. Some of the participants, particularly the elderly ones, reported that they had tried and left different forms of business before their current undertakings.

**Table I:** Respondents’ demographics and business profile.

Participants	Item traded or kind of vendor	Age	Years in marriage	No of dependent children (family size)	Years in business	Estimated daily earnings (in Naira)
R1	Clothing, bed sheet, curtains	40	12 years	3 (5)	3	2000–10,000
R2	Hairstylist	31	6 months	0 (4)	3	500–2000
R3	Food vendor	38	6 years	0 (2)	4	5000–10,000
R4	Fruits and vegetables	34	6 years	2 (4)	4	800–2000
R5	Baking materials	38	15 years	6 (7)	3	8000–15,000
R6	Beddings	35	9 years	4 (10)	2	5000–15,000
R7	Food stuff and fruits	45	25 years (widow)	6 (7)	15	5000–10,000
R8	Hairstylist and food items	35	8 years	4 (10)	18	100–10,000
R9	Groundnuts, popcorn, sugar, and soybeans	40	23 years	6 (9)	7	200–500
R10	Food stuffs	35	11 years	4 (8)	6	1000–4000
R11	Vegetables	30	12 years	4 (9)	8	1000–2500
R12	Nuts	40	26 years	7 (10)	20	5000–10,000
R13	Vegetables	41	23 years	3 (5)	15	1000–1800
R14	Pepper	40	17 years	3 (6)	19	3000–4000
R15	Pepper	37	7 years	2 (5)	6	1000–3000
R16	Vegetables	58	33 years	9 (8)	30	2000–7000
R17	Fish	30	10 years	5 (7)	5	5000–10,000
R18	Food stuffs	48	31 years	6 (8)	10	500–1000
R19	Food stuffs	34	7 years	3 (6)	9	4000–10,000
R20	Food stuffs	45	34 years	6 (8)	15	2000–5000

R, respondent.

Background information, regarding the respondents’ age, marital status, family size, and queries around their businesses were taken before the commencement of the main interviews. The participants indicated having been in their trade for a range of 2 to 17 years. They put their gross daily income to range between N100 and N15,000 (naira) (using current dollar exchange rates, $0.41 to $62.98), placing them in the low-income range.

### Data collection

A semi-structured interview schedule was prepared and deployed by the researchers. This is in line with the observation that in-depth semi-structured interviews are the most common method of data collection in qualitative studies (Brooks, 2015; Smith & Osborn, 2008). Interview questions included 12 questions that were a mixture of open and close-ended questions. The interview schedule was constructed and its items vetted by all the researchers. These questions were then translated and back-translated in the local language (Igbo) by a professional linguist. The interviews were anchored on the three broad themes of conceptions of business-life balance, interference between business and other life roles, and coping and motivating factors in managing business and life roles. A brief description of the topic of interest was explained to the respondents before commencing each interview. This was particularly important as it had been noted in a pilot scheme that many women struggled to make sense of the concept of work/business-life balance, partly because the concept was new to them, and the vernacular transcription for balance is not commonly used in the everyday discussion of business and other life roles. Prompts and probes were used to elicit more detailed information regarding issues deemed important by the interviewer or raised by the respondents.

### Procedure

Following the approval of the Department of Psychology ethics committee, the women petty traders were approached to participate in the study. Twenty-six consenting volunteers were drafted, and the day and time for the interview was scheduled. Inclusion criteria included being married and running a small business as well as having a family. Twenty women were, however, available and indicated readiness on the scheduled day. Participants sometimes required a further breakdown of the concept, with examples of illustrative scenarios, before they could begin weighing in on the discussion. All the respondents preferred and used the Igbo language in responding to the interview questions. It was, however, not uncommon to find respondents mixing up the local language and English in trying to express their views and opinions. Since respondents had some difficulty grasping the concept of balance in the context of business and other life roles, instances were given using specific family roles and business roles (e.g. What is it like covering the market and doing home chores or school runs?). Participants would normally tend to stick with the business-family example in relating their experiences. The interviewer therefore needed to repeatedly remind them that the scope is not limited to their families and to highlight some other activities common in the locality (for example, religious and funeral obligations). Interviewers, most times, used probing statements. The interview was recorded electronically with the consent of the respondents. These recordings were tagged with numbers and stored in a password-secured computer. Responses were later transcribed and translated for interpretation by a professional linguist who was paid for his services. He understood and agreed to the research ethics.

### Data analysis

We adopted Interpretative Phenomenological Analysis (IPA, Smith, 1996; Smith & Osborn, 2008) in analysing the transcript. Phenomenological research focuses on the life-world, the lived personal experiences of the respondents, by getting into their personal and social worlds, and laying bare how daily experiences are conceived in the conscious mind (Smith & Osborn, 2008). The first and second authors (DU & CO), who were a bit more experienced in qualitative research, followed Smith and Osborne's (2007) recommendation to look for themes and interpretation. Smith and Osborne suggested reading and re-reading the transcripts for the first few chosen cases, noting salient points made by the respondents and writing down themes. These practices improved familiarization with the data. DU & CO also listened, over and over, to the audio recordings of these cases, which were chosen from the cohort, commented on them, and identified themes. Listening to the recordings repeatedly was important to data immersion, an important ingredient in qualitative research. Repeated listening often provides cues (such as pitch and timbre), that may be omitted if transcripts alone are used. The identified themes were further clustered, seeking linkages and order to reflect a theory consistent with the concepts, especially as the theory relates to work-life literature. The interview guide had been structured to explore different dimensions of interest and eased the categorization of themes from the transcripts. Subsequently, all authors joined in screening other transcripts feeding into the identified themes but keeping an eye out for new distinct themes. The final outcome was re-read and agreed upon by all the authors.

## Results

### Overview of the data

Among the 20 participants, 19 were still married and lived with their families and one was a widow. Table I (below) shows the respondents and their demographic composition.

### Findings from interview

The three-pronged nature of our inquiry received considerable details during the interviews. First, in conceiving business-life balance, there seems to be a thin line between business and other life roles, hence women talked more of “my roles” or “life roles” without necessarily distinguishing between defined roles. Though discussions of balance largely reflect flow and rhythm across roles, themes of effectiveness and satisfactory progress were used in describing balance. However, the ability to navigate the stress and difficulty in adequately fulfilling one's duties, roles, and obligations stood central in their experiences. Interestingly, these difficulties are perceived and communicated as being expected and more or less acceptable as part of the roles of a “married mother,” which provided a form of buffer to the overwhelming feelings of stress reported.

Experiences of distress, sense of loss, the burden of extra-social roles, and anxiety featured in the overlap of business and other life roles. Acceptable distress was, however, at the core of the experiences of the women when they pondered how business roles interfere with other roles and vice-versa, highlighting the traditionality of their conceptions of their roles. Helpful figures, lifestyle, and motivators studded their coping mechanisms to the stress of business-life. We present these reports in line with the broad three areas structured in the interview guide which were conceptions of business-life balance, interference in business, and other life roles and coping with business and other life stress.

### Conceptions of business-life balance

Most of the respondents appeared to struggle with the concept of business-life balance. They understand, for example, that family roles are different from business roles and give appropriate examples of both roles. The concept of balance, however, was understood from diverse viewpoints. It was interpreted by some of the participants to mean satisfactory progress in all roles that one may indulge, others considered time rationing in their descriptions and yet some others construed balance as harmony, synchrony and/or rhythm. A deeper look in their reports highlights that both roles are not considered to be essentially distinct; the women saw these roles as those of married women, in a sense, life roles, and so do not tend to consider the stress coming from these roles as unexpected or unusual as demonstrated in the quotes:

… what I understand how? It is like getting my home in order and at the same time getting what my shop needs … it is something that I must be doing. (R1)

… that is, you see, you mean that I cannot be doing this one and that one at the same time. Because they [business and family] are not together, it is not one, it doesn't fall in place, there is no matching, flow, in their combination … but they are my roles. (R7)

I understand it to mean that I have to be successful in both places. That I do all that I am supposed to do in the business place and all I am supposed to do in the family. (R5)

The women's descriptions of business-life balance are akin to those portrayed in the work-life literature. Themes such as equal attention (Marks & MacDermid, 1996), satisfaction across roles (Clark, 2000), and rhythm and harmony (Munn, 2009), were reflected in their description of business-life balance. The latter theme of rhythm and harmony illuminates the level of personalization with which the women regard their roles. They conceive business-life roles as their own roles and so make no clear-cut distinction between business roles and other life roles. This seamless connection between business-life roles may be considered somewhat unique. The result thus shows that there is a large similarity but maybe subtle differences in how balance is conceived by business and employed people, and as well people in different economic classes and cultures.

At the core of the women's description of balance, is the issue of addressing the difficulties/stress arising from multiple roles. The pursuit of balance in the business-life sphere for the women is essentially how to effectively deal with the expected stress arising from their daily duties with the resources available. Many women point to the physical stress, strain, and difficulty in discussing business-life balance:

… it tears the body [me] apart, but it won't prevent eating in my family, it does …, ehh that you are [I am] in business doesn't prevent cooking in my home. (R4)

… It bites me in the heart … it disturbs and disturbs very well as my mind is in the home and my mind is in the market … I am not even sure I get it right in both places, sometimes. (R14)

These quotes indicate that the feeling of discomfort holds a special place in the women's perception of their struggle to attain balance. From these conceptions of business-life balance, it can be discerned that attaining balance for these women implies the effective dissolution of stress and the difficulty experienced in carrying out roles. However, there were unique variations. Some participants had a different perspective of balance. Balance in business and other life roles were thought by some women to be attainable only when they can draw on one or more other helping hands. So, in some sense, they consider an individual's attainment of balance to be nearly impossible without external hands lending support. Some identified their partners (husbands), holiday makers,^1^ relatives, neighbours (at home or in the market place), and house-helps as possible means of attaining balance:

Just like this time we are entering into the holiday period, I am expecting that some people will come around. This person will be handling, especially people with strong hands, so that … I can be coming on time to the business, this time of holiday. But during school days, I will have to carry on myself, cover the shop, and get my children (R6) and

I understand it to mean … eh, if things are good for you, if like, say you have a helping hand, then both roles will be going fine and will not strain you much … if the help is there … eh, then you'll relax and do one. But if it is only you, then you'll be really stressed to get these things in order; you cannot be here and there (R4).

The above quotes demonstrate conditional balance, a conception that attaining balance depends on having an external helping hand. However, a salient point seeps through. These quotes showcase how socio-economic status impacts the conception and pursuit of balance. Most women who view balance in a conditional way appear to be a little better off financially or otherwise. Either they have a partner (husband) who have attained a moderate socio-economic status (at least within the locale) and are therefore able to attract and host holiday makers and/or hire house-helps, or they are slightly older adults who live with teenagers/adults (mostly females, as is always mentioned) that perform a large chunk of the family roles. Each of these groups of women reported less distress, compared to that reported by other groups, when asked to consider their level of distress and their ability to juggle business and other life roles.

What seems to be at play is that though most of the women report stressful experiences, women of lower economic status appear to have limited resources to assist them (typically only their husbands), and so have less capacity to take on demands in their different roles and so attain a more personalized view of their roles. On the other hand, women with better socio-economic status are able to seek extra hands to assist with (increasing) routines and roles and so have less pressure from these roles and probably better wellbeing. However, women across the board largely accepted their stressful experiences as part of everyday living in the culture, demonstrating the significant level of social influence in how they conceive and pursue balance. But the impact of the wider social background in their description of what business-life balance could mean can be seen in a sub-theme called norm/helplessness.

Total acceptance along with a helpless feeling was a noteworthy find; the constant gesture that there is nothing “one” can do about “it.” Many of the respondents reported that performing business and family roles, amidst the difficulties, is part of everyday living and that “nothing can be done” about it as both roles are considered important. This consideration was reiterated by most of the participants who would normally end their description of business-life balance with this gesture. It is stressed so much that it appears the women have learned to be helpless about the situation and carry on with their daily routines, attaining almost a state of personalization of both roles.

… it will be good to [get these roles properly attended to], it is difficult to get both OK. But … what can I do … it is what I must do … I have to do it. They are roles meant for me. (R9)

Well, even if my family roles affect my business, what can I do? I can't stay idle, I will have to finish my deals in the home before coming to the market. (R18)

These quotes demonstrate the stance of these women regarding the stress they go through in pursuing balance. They are quick to identify these struggles as a normal part of living and therefore do not seem to consider the issue of balance to be of particular importance. This integral view of business-life roles as a normal flow of life appears to rather downplay the stressful experiences. Perhaps because these roles have come to be largely commonplace in the culture milieu, the adverse effects on wellbeing may be minimized.

### Interference of business with other life roles

Women reported how they are caught between business engagements and other life roles, generating pressure that describes the source of the distress reported by women petty traders. Consequently, the theme of interference highlights negative reports on the physical and mental wellbeing of the traders. Family roles, deemed more important, most times, were referred to as the chief source of interference with business engagements. Most of the women highlighted that it does not feel easy for them to combine both roles.

It bites me in the heart … it disturbs and disturbs very well … my mind is in the home and my mind is in the market …. I am not even sure I get it right in both places, sometimes (R14)

I don't get myself … I get distressing calls from home when I am in the market to tell me that this my child is sick or that they have not come back from school and it is getting late. You just get tired and ask yourself what you are doing there [in the market] … you are no longer yourself, you keep calling …. It can be distressing. (R18)

The highly dramatic description of distressing somatic and mental experiences of stress highlights the difficulty experienced by these women in their daily activities. It may also be noted that, being engulfed in this stress, one develops some sense of self-doubt, and questions the rationality of keeping on with a current engagement. The women were caught in a hard place between weighing the costs and benefits of a particular engagement. This feeling draws attention to another widely reported experience—a sense of loss.

Many respondents report abandoning business commitments to meet family demands and vice-versa, generating a feeling of loss in either the family or business place. They report closing up their businesses to attend to pressing family roles but not without a feeling that they must have missed out on something, mostly sales in their business place.

… in the morning, I have to prepare the kids to go to school, prepare their meals and after doing all these things and getting to the market, you will see that customers have bought their goods and have since gone. So you do not make much at the end of the day. (R15)

I don't feel relaxed when I know I am losing some of my produce to rot. I am at home but my mind will be on the produce I have heaped in there [in the market place], it worries me. (R19)

I feel mostly affected when I consider how combining my business and family roles affects my children. I find that I don't give enough time in their education, you know, help them with assignments …. I find a teacher to help with lessons, but you know, My mind is not at peace, I still want to know how they are doing. (R6)

These quotes illustrate that the loss accrued from turning attention to other roles may be financial (Rs 15 and 19) or emotional (R6), and generates critical self-evaluation and feelings of guilt. These feelings will add up to the distress experienced by the women.

But family affairs are not the only engagements that cut into business activities. Perhaps more striking are resource-demanding engagements in extra-social roles that are peculiar to the local culture. Respondents raised concerns about the interference emanating from them having to attend to socio-cultural and religious obligations. Issues arise concerning burial rites, religious commitments, and marriage events, which are highly valued social events within the locale, but are also activities that grind into business engagements. Some report attending these social roles not out of direct volition but to meet social expectations and ensure that their participation will be reciprocated when their own “rainy day” comes and they must host such events. Nonetheless, they reported that attending to these obligations was a large source of distraction and stress, especially for business roles.

… it is very disturbing. You may just come back carrying your [new] food stock and you get the news of the death of someone. You just abandon everything and lock up everywhere and go to it. My children cannot leave what they are doing to come to the business. The fruits, who cares? If it is spoiling, it is not my concern, because that dead person is greater than fruits, even though the person is dead. (R16)

… you know when death happens, you will have to close up your shop. If it comes to a brother or sister, you know an insider, a close relative, ehh, you can count one month off. (R9)

Attending to these extra-social roles and obligations includes financial commitments and a suspension of business activities, adding to the pressure the traders face, given their already meagre incomes. However, to comply with social norms, they conform to the demands that the situations require. To demonstrate how difficult it is for these women, one woman reported a state of latent anxiety upon receiving calls from the carer of her elderly mum:

The other day I was called [on the phone] and was invited for a talk after market^2^ [business], in the evening. I hurriedly called back and asked her to tell me now. Because I have an elderly mum who is very old like this woman sitting over there. My mind went to her and I asked if I should start coming [thinking she has passed on]. She said no that she was asking after us because it has been a while since she heard from us. (Int: so you got upset?). Yes, I remember saying to myself [*pauses*] this old woman should not die now [*pauses*] because there is no money. (R16)

Anticipating the death of an elderly relative, a parent for instance, evokes strong uneasy feelings that are, possibly, a combination of depression and anxiety. Though depressive feelings may result from the anticipated loss of a loved one, feelings of anxiety pop up as an issue for the trading women regarding the financial demands from culture-based societal obligations. In the case of the woman mentioned above, anxiety arose from the heavy financial burden of the burial rites of her mother. This feeling put her into some sort of pre-traumatic state, which could be traced to the expectation of overwhelming distress. These external anxiety-provoking expectations, often associated with financial demands, impact concentration, especially in the business place. Respondents frequently made reference to receiving phone calls that require them to attend to social, religious, and family functions. The most severe reported distresses were for sick children and relatives who have just passed on.

### Coping with business and other life roles to attain wellbeing

In exploring what the women do to carry on with business and other roles, respondents raised quite an array of approaches with which they cope with their roles. Approaches to coping with daily living in the business-life sphere were three-pronged: who helps, effective strategies, and what motivates. Subthemes identified include the following.

#### Helpful figures

Participants refer to a number of figures as helping them get through the daily hassles of their roles. Many of the respondents indicated that their husbands’ support really matters and helps them. They recognized an understanding and supportive partner to be helpful in putting up with the business and other life roles. Most admit that, even though they do not expect their husbands to play certain roles, their husbands clearly partake in doing some home chores and caring for children. Others had assistance in business and family roles from grown-up children (mostly teenagers), mother-in-laws, and neighbours in the business place who cover for the women and make sales when an individual is out to attend to other needs; neighbours at home who agree to look after children as the women attend to the business place; holiday makers who cushions the stress by performing home chores and sometimes come out to the business place; extended relatives who agree to cover social obligations; and even children who would help especially during the holiday, in both home chores and in the business place.

… if I am around, he may not bother with some things, but if I am not there, he surely helps, if there is food, he cooks, … say I have been away and came back tired or something like that, he can help with the things he can … (R4)

My husband helps a lot. (How? What things does he do for you?). *Laughs* … he takes care of the children, and once he is the one there I feel relaxed and don't worry about them while in business. (R17)

… if I know I won't be around to take care of things, I will just give my key to this my neighbour here [*points to the neighbour*]. She will display my goods … At times when a customer makes a bargain that I don't like and I scold the person, if she sees that I have a little profit on it, she will walk over, call the person back, make the sales and *dump* me my money, [*laughs*]. … at home, I do not have to cook, my children are grown up, I relax, they do the cooking and will keep doing it until they all get married. My clothes, ah, sometimes I wash, sometimes they carry everything and wash them clean. (R16)

From these interview extracts, an immerse network of social support figures are available to the women and are helpful in performing their roles. Positive feelings (expressed in laughter) hint on the feelings of relief offered by the supportive figures. This experience corroborates the findings of other researchers who identify supportive family figures and colleagues as being helpful in maintaining a better synergy between work and family life (Bakker, Schaufeli, Leiter, & Tarris, 2008; Lu, Siu, Spector, & Shi, 2009). Beyond the existence of this support network, the honest perception that these supportive figures are helpful has been reported to be important in alleviating the stress of working people (Ruikar & Abhyankar, 2015). This positive perception will be most helpful to the trading women, giving them relief from the stress and difficulty they experience. Again, the reference to husbands who are active helpers in family roles (e.g. in cooking and child care) hints that there may be a shift in beliefs and adherence to traditional gender roles, even in rural traditional or collectivist societies in which traditional gender roles are still largely upheld. Perhaps the support and assistance given by family figures explain why family roles are ranked at the top among the roles of the women, elevating the pre-eminence of the family as a strategy of adapting.

#### Lifestyle approaches and strategies

Respondents shared a strong consensus on the eminence of family roles over the business and perhaps other life roles. They reported foregoing business roles to attend to family issues that they consider, at the time, to be pressing. The priority given to the family appears to enable them make quick decisions as to what matters most. With the family pre-eminence, it is easier for the business women to switch roles, sparing them the mental task of contemplating the relative importance of an activity.

Sometimes they keep calling me here [on the phone] to come and attend to customers, I just ignore them and keep on with what I am doing [attending to the family]. (R5)

Even if I have a demand, you know someone wants to buy something big, and maybe say my child is sick or something like that [shrugs], I will close my shop and attend to my family. Because, even this shop should come last. (R6)

The interview excerpts suggest that petty traders could abandon the benefits of the business place, including making sales to attend to family roles. The availability of such options, on one hand, point to the control and flexibility available to self-employed people, but on the other hand, and more important for us in this context, it suggests a clear stand of placing the family first in the business-life sphere. This finding is consistent with work-life literature research that suggests that other roles revolve around the family role.

Extending the importance and regard accorded to family roles, a salient approach to coping may be seen in the prioritization of roles based on their relative perceived importance. One interesting approach reported by the women was to weigh the relative importance of roles as they come by and its relative concern to an individual:

You know, it doesn't feel to me that my business will knock out my family affairs. Because before I come to the market I'll see how my children are doing in the morning. I don't wake up early in the morning to rush out to the market. I and my children will exchange greetings … each of my children. Like yesterday they will be having extra-lessons so I have to prepare the meals they will take to that, get them ready, see that they are off to school, this one is first. … when I want to attend burials, the market will close … I am not two persons, I'm just one. (R12)

The way it is for me, my neighbours would cover for me in the market if I'm not around, but in my home, there's no body, because I am not staying with any other person that can help. So if you know what is more important for you, you face it. Sometimes it will mean you will not come to the market. (R13)

It has been suggested that being able to determine what is important in one's life is helpful in navigating everyday life, just as prioritizing can be effective in reducing the experiences of stress (De Ridder & De Wit, 2006; Versey, 2015).

Effective time management through early planning and organization is yet another strategy deployed by the business women in ensuring balance across roles. Many of the respondents had an implicit schedule for when to attend to specific roles and when to switch to some other activity. Apportioning time to source produce, do home chores, and attend to the education needs of the children were common on the reports:

… there is suffering in it. But I wake up by 4 or 5 to get the children ready for school. In the days they have extra lessons, then you will have to start even earlier to prepare their meals, take them to school, before I come back to sweep the house and prepare for the market. (R6)

… it is not easy but I have to spread and share it out … (Int: So you share it out?) … yes, it is shared. … in some days, I have to get into the bush market [more interior rural areas] to source for produce … So I have learnt to set aside a day … or two in a week, leave the market and work in my home so that when I come home, I can find a place for my feet … (R14)

Conscious planning and being proactive in approaching roles underscore time management as a strategy of navigating between business and life roles.

#### Motivational

The financial benefits that come from engaging in business were noted to be hugely motivating and spurred these women to carry on against all odds. To some, the hope for high sales and financial proceeds were strong motivating reasons to tolerate the stress of business life.

R6: I find happiness to carry on with both roles because I know that I will make money to attain to the needs of my children and family …. I don't have to keep asking my husband for money ….

R7: … you know our people say that wealth strengthens the heart … if money is entering your hands, you will be happy. If you have problems, you can see money to help you to solve them … yes, you will be getting yourself.

The quote “wealth strengthens the heart” is a literal translation of a local saying that implies that “wealth breeds confidence” and demonstrates how much important finance is for the petty traders. Optimism for financial benefits and the actual financial benefits from the business place could be understood to enhance feelings of wellbeing and self-worth, and a more positive self-concept in these women. The financial gains accruing from the business sphere go further to generate a feeling of satisfaction as they help in other life spheres, especially in the family.

The satisfaction drawn from seeing both home and business flourish was enough for some women to focus less on their own negative distressing experiences. Some of the respondents reported that they have come to enjoy their roles, particularly when they consider their business and family domains satisfactory:

I am happy, yes … I run around a lot but I am OK with it. I will go from the home to the market and back, and when I see that my home is good and my market is also good or that I am able to use money to take care of everybody I am supposed to take care of, children, husband, and parents, I will not remember the difficult times. You can't take care of people empty-handed. (R15)

This excerpt points to satisfaction across roles but also points out the positive spill-over effects across roles. Resources acquired from the business help the women assist themselves and their families, invariably rendering a feeling of fulfilment and satisfaction. Though spill-over effects may be either positive or negative, Greenhaus and Powell (2006) highlight the benefits of facilitation, role integration, and enrichment of positive spill-overs.

Last, some of the respondents found hope in anchoring on their religious beliefs. They reported that their belief in God sees them through the stress of family and business.

My Jesus is always close every time, giving me good health to carry on. (R10)

It is difficult, you see …, I can't imagine that anybody can perfectly attend to everything he has to do. We just count on God … you do your best and leave the rest to Him. (R1)

Once I remember that God is there, you know put my hope in Him, once it is in my mind, whether I have completed my home duties or not, whether I did fine in the market or not, I will just be happy … I don't remember the worries in my home or in what shape my shop is. (R12)

## Discussion

We examined the experiences of petty traders regarding business-life balance in a LMIC where most people are self-employed and have little or no access to a welfare system. Their experiences were observed to share similarities with issues consistently raised in many Euro-American work-life studies, but also had fairly unique conceptions and interpretations. Though satisfaction across roles and equal attention resonated with shared meanings in popular literature (e.g. Clark, 2000; Greenhaus et al., 2003; Kirchmeyer, 2000), descriptions of balance as harmony, flow, and rhythm apparently assumed extended unique meanings. These latter opinions align with the ideas of McMillan et al. (2011) in work-life harmony and Munn (2013) that opined a work-life system. This finding suggests that business and other life roles for these women are thinly, if at all, separated. They appeared to see all roles as life roles. Their integral view of these roles seems to flow from their personalization of these roles, such that it appears that being married and having a family demand that the woman be actively engaged in a business as well as meaningfully attend to other life roles as expected. This systemic view of life roles aligns with the projections of more recent work-life researchers who consider the work-life spheres a system (Munn, 2013). What transpires in between these roles is considered in terms of synergy and harmony (McMillan et al., 2011). Additionally, many women considered balance to be conditional, and attainable only if one has a helping hand. This corroborates the findings of Rehman and Roomi (2012), who found spousal support to be a substantial consideration by Pakistani women in defining balance. Though this finding may seem distinct from a conceptual definition, it highlights the importance of external and/or significant others in facilitating the attainment of balance, at least for women in business who earn low incomes. Expanding on this, business-life and work-life literature may be understood to be beyond the experience of given individuals, as this research highlights how other family/community members are drawn into the dissolution of the stress accumulating from business-life roles. Perhaps, work-life literature needs to turn to exploring how the pursuit of balance involves others in a communal system.

In examining how issues in business interfere with other life roles and vice versa, respondents highlighted the difficulties arising from the culminating pressure of striving to attend to roles in different spheres. The women reported physical and mental distress, a disturbing sense of loss, and anxiety-provoking expectations when they reflected on the challenges in playing business and other life roles. These negative experiences were expected as they are commonly reflected in literature in people who engage in multiple roles (Greenhaus et al., 2003). The slight but interesting difference was the contingent waiving of these stressful experiences upon acknowledging their existence. It appeared that these experiences were in a way expected and accepted. An explanation could be that the expectations of womanhood and motherhood, at least within the cultural context, require some form of doggedness and resilience. This could be explained by Lyness and Judiesch's (2014) cross-cultural observation and suggestion that women's (but not men's) appraisal of work-life balance is context-dependent, influenced largely by the culture. Helping these women in their appraisal of their experiences and expectations may involve helping them find meaning and relevance for their businesses and other role engagements as meaning in work has been found to be effective in workers (Munn, 2013). The impact of conflicting business-life roles may be understood, therefore to be somewhat influenced by the individual's personal appraisal of her or his experiences, which may not be far from what is upheld in the cultural circle.

Respondents reported a desire to have all roles sufficiently attended to and identified how they go about pursuing balance. Identified influences that spurred them on clustered around helpful hands and effective lifestyle approaches/strategies. Husbands and a mix of adjuvant support persons (e.g. neighbours in the home and in the business place, close and distant relatives, holiday makers) in the home and the business place were identified as being helpful in easing the stress of business and other life roles. This demonstrates the characteristic support system in the typical African setting that, in many ways, stands in for the absence or grossly limited capacity of a welfare system. However, as mentioned earlier, Rehman and Roomi (2012) found husbands’ support to be essential for Pakistani women. Effective strategies and approaches identified and commonly deployed by the women to attain balance and ensure wellbeing included placing the family first, prioritizing demands, and managing time by planning and organizing to attain balance and ensure wellbeing. This highlights that individuals could devise strategies that are personally effective in ensuring wellbeing and balance in life roles. This has been corroborated in other findings (e.g. Greenhaus & Powell, 2006; Zheng et al., 2015). Also, their optimism about financial gains, recalling the satisfying state of affairs across life roles, and a hope in God, provided the motivation to carry on in performing life roles. This approach to the pursuit of balance and wellbeing has interesting undertones.

First, being a region largely oriented to traditional gender roles, it is surprising to find that men were reported to be willing and actually played some traditional female roles identified as cooking and child care. The possible adjustment in traditional roles underscores a positive note for the women, as they can at least find relief as men take on some traditional feminine roles. This may also reflect the complementary role of males considering that their wives are equally playing bread-winning roles. As much as we consider this a credit for the women and their partners, we do not have enough data to claim that women who reported that their husbands willingly help in home chores are better off in health and wellbeing compared to those whose partners decline traditional home roles. Comparing the wellbeing of individuals who subscribe to traditional roles with those who downplay those roles would make an interesting pursuit for future research in the population studied. Second, the primacy of family roles may be understood to help the women anchor the essence of their struggles, to preserve and ensure the progress of the family. This tends to spare the women of the mental cost of deciphering which roles deserve more attention and resources. In another light, however, prioritizing family roles may be another signature of traditional family roles as the women may still consider family roles and demands to be their primary roles, hence all other roles are subordinate and could be sacrificed for family roles.

Yet from another perspective, conceptions of business-life balance match the view of Khallash and Kruise (2012) that work/business is an instrumental element, “a means to support a way of life and to create optimal living conditions for one's family and/or oneself.” Third, prioritizing roles and demands can be seen to be of help to the women in forming a mental hierarchy of the importance and urgency of diverse roles and engagements (especially roles outside of the family). The theme of priority equally aligns with Munn's (2013) definition of the work-life balance regarding balance as reflecting how individuals choose to prioritize their work, family, individual, and societal commitments. This reaffirms the idea that what is important for individuals in attaining balance is relative and personal. The danger in viewing balance this way is that it could be understood to invariably suggest that “nothing works” or putting it defiantly, “nothing will work,” because balance means whatever is situationally important to individuals in a given time and place. Consequently, government and organizations’ commitment to implement measures that will ensure a healthy and well-fit population, including employees, would ultimately be in vain if balance is what individuals consider a priority at a particular time.

On issues of motivation, the respondents’ optimism for financial gains from their business roles was largely inspiring in pursuing life roles. Again, these benefits were largely identified to be deployed in meeting family needs but were equally hinted to be important in meeting social and community obligations. This highlights Greenhaus and Powell's (2006) enhancement theory that the benefits of diverse roles spill over to positively impact other domains. Other researchers have equally found financial gains from the business place or entrepreneurial activities to be highly motivating for women (Bull & Mittelmark, 2009; Rehman & Roomi, 2012).

However, the extent to which the concept of work-life balance captures the peculiarities of many African settings is yet to be clarified and can only be understood with an increase in work/business-life studies. We contend that socio-religious expectations and the commitments demanded in many African settings can be onerous and therefore demand closer scrutiny. We therefore advocate that well-structured research is needed to weigh these extra-social roles, examining whether they are substantially abstracted from family roles; are uniquely different from the social experiences and engagements of people in other cultures; and whether they considerably compound the stress of people in the region. This can be approached using either quantitative or qualitative methods, examining the relationship between social engagements and experienced stress and wellbeing. Our hunch is that the relationship would be complex because diverse social relationships offer both enrichment but can also create stress, depending on the dynamics of the degree of involvement, personality types, and contextual values placed upon those engagements. Bull and Mittelmark's (2009) study of mothers in the Scandinavia hinted that social participation was found to be related to wellbeing in non-single mothers and that lower social participation matches lower wellbeing scores. Further exploration is nonetheless important as a number of complexities may be in play in the situation. For instance, in burial rites, closer relatives are expected to stay in the ceremony for days while distant relatives may spend fewer days. In-laws, friends to the children, and siblings of the deceased are also linked in burial rites as they are obliged to display solidarity by attending the rites. In a Nigerian setting, it is not uncommon, even in the public sector, for individuals to take a day off work to attend to a colleagues’ relative's funeral.

### Limitations, contributions, and directions for future research

Aside from the small sample size and the general problems of subjectivity and non–generalizability associated with the use of interviews and other qualitative approaches, a number of issues were of concern to us. We admit the possibility of loss in meaning in the process of translation, transcription, and re-translation. We nevertheless tried to reduce this loss by deploying the services of a professional linguist. Also, the lead author (DU) and lead interviewer (LU) were natives of the local community who spoke and understood the local dialect and were closely involved in the formulation of interview schedule, interviewing, and coding the transcript.

Again, the approach of interviewing the participants in their business spots posed little challenges. Though this approach gives authenticity to the research, there were (in two cases) interruptions by customers trying to purchase goods from the respondents or the sound of vehicles passing by. Each time this happened, the interview process was paused and later continued when the respondent collected herself and indicated a willingness to carry on. It may be argued that a disturbance in thought processes may be implied, but there was no reason for us to surmise that any of the respondents strayed very much from the subject of discussion. Marshall and Rossman (1999) have suggested that studies should be carried out in the setting where the phenomenon of interest occurs. Again, in isolating themes and coding the transcript, we found no fundamental disruption or distortions in these women's reports that could be attributed to interruptions.

We largely mulled over adopting IPA over thematic analysis in synthesizing the interview transcript, especially when we considered the deep level of language and idiomatic languages that respondents deployed in communicating their views. Nonetheless, we stuck with the two, considering IPA first, to allow for little bias given broad knowledge of the topic and subsequently using thematic analysis to identify and distinguish themes.

Notwithstanding the above limitations, this study, to our best knowledge is the first to qualitatively explore the experiences of women who are small-scale traders in the Sub-Saharan region. The findings showed that work/business-life balance is likely to be perceived relatively similarly across resource-earning engagements, governments, and cultures. Difficulties emanating from work and other life demands are not only experienced by employees in formal organizations or “typical” entrepreneurs in their businesses; these experiences are shared equally by petty traders who earn low incomes. More important, the findings challenged the widely held bi-directional conceptualization of work/business-family conflict. In most cases, participants in this study were equally concerned with the intrusion of social engagements into the business domain, and vice-versa.

This research identified a number of social and community engagements that were widely reported by the women. The scope of this research gave little chance for a deeper probe of the distressing and supportive dynamics that these social engagements offer. Future research may specifically examine what these social engagements, especially culturally oriented practices that are largely around traditional ceremonies and burial rites, may imply and impact balance in both business/work and other life roles. With women being the focus in this study, future studies could pay attention to men who engage in small businesses and earn low incomes in the region, exploring their experiences and conceptions regarding how to balance their business and other life roles.

## Conclusion

We conclude that the experiences of petty traders in the LMIC studied largely align with reports in the work-life literature, though slight differences exist in the seemingly more integral view of business and other life roles, with family roles taking eminence. Informal relationships in the African context are highly cherished and present a unique replacement for the deficient welfare system and policies that are almost non-existent work-life balance measures, particularly for low-earners. The social demands and social commitment in the sample studied are quite high and impact wellbeing. However, there is little understanding regarding the nature of these commitments. A cost-benefit analysis and comparative study of high commitment and low commitment in social engagement is needed to understand its dynamics across cultures. We, therefore, strongly suggest it as a unique front in the work-life literature. Local and international policies aiming to impact low-earners may focus on the education and health care needs of children and the elderly to ease the pressure on the meagre income level of this category. However, community and familial support seem to be highly cohesive in the region, hence programs that foster community cohesion and integration should be encouraged in the areas. Future studies should look at the experiences of more women as well as men in similar settings. At least for the population we studied, priority seems to be placed more in the family roles, particularly the welfare and wellbeing of family members.

## Notes

1 By holiday makers, we refer to individuals (mostly children and adolescents) who, during the holiday periods, travel to be with their relatives or family-friends living in other parts of the country.

2 “Market” is used in the local language to refer to the market place, sales, business establishment, as well as business transaction.
